# Beyond the whole genome consensus: Unravelling of PRRSV phylogenomics using next generation sequencing technologies

**DOI:** 10.1016/j.virusres.2014.10.004

**Published:** 2014-12-19

**Authors:** Zen H. Lu, Alan L. Archibald, Tahar Ait-Ali

**Affiliations:** The Roslin Institute and Royal (Dick) School of Veterinary Studies, University of Edinburgh, Easter Bush, EH25 9RG Midlothian, United Kingdom

**Keywords:** PRRSV, Ultra-deep next generation sequencing, Phylogenomics, Quasispecies

## Abstract

The highly heterogeneous porcine reproductive and respiratory syndrome virus (PRRSV) is the causative agent responsible for an economically important pig disease with the characteristic symptoms of reproductive losses in breeding sows and respiratory illnesses in young piglets. The virus can be broadly divided into the European and North American-like genotype 1 and 2 respectively. In addition to this intra-strains variability, the impact of coexisting viral quasispecies on disease development has recently gained much attention; owing very much to the advent of the next-generation sequencing (NGS) technologies. Genomic data produced from the massive sequencing capacities of NGS have enabled the study of PRRSV at an unprecedented rate and details. Unlike conventional sequencing methods which require knowledge of conserved regions, NGS allows *de novo* assembly of the full viral genomes. Evolutionary variations gained from different genotypic strains provide valuable insights into functionally important regions of the virus. Together with the advancement of sophisticated bioinformatics tools, ultra-deep NGS technologies make the detection of low frequency co-evolving quasispecies possible. This short review gives an overview, including a proposed workflow, on the use of NGS to explore the genetic diversity of PRRSV at both macro- and micro-evolutionary levels.

## Highlights

• NGS allows the whole genome sequencing of PRRSV without any prior knowledge.
• Low frequency variants within the co-evolving quasispecies can be detected.
• Both macro- and micro-evolutionary events can be followed using NGS.

## 1 Introduction

Porcine reproductive and respiratory syndrome (PRRS) is a devastating disease resulting in huge economic losses to the swine industry worldwide (Neumann et al., 2005; Nieuwenhuis et al., 2012). The disease is characterised by late term reproductive failure in sows and respiratory distress in young growing pigs (Wensvoort et al., 1991; Zimmerman et al., 1997). Its viral etiologic agent, PRRS virus (PRRSV) of the family *Arteriviridae* and order *Nidovirales*, has been found to exist ubiquitously in almost all affected swine populations (Brockmeier et al., 2012; Shi et al., 2010). The positive-sense RNA viral genome is approximately 15 kb and it encodes 14 non-structural and eight structural proteins in eight open reading frames (Meulenberg et al., 1993; Snijder and Meulenberg, 1998). The virus has a narrow cell tropism with a preference for cells of the monocyte/macrophage lineage infecting specific subsets of differentiated macrophages in lungs (*i.e.* alveolar macrophages), lymphoid tissues and placenta (Snijder et al., 2013). The evidence on PRRSV binding, internalisation and genome release has been reviewed elsewhere and a model of viral entry proposed (Van Breedam et al., 2010). Briefly, attachment of PRRSV to target cells is mediated by the interaction between viral glycoprotein 5 (GP5) and porcine sialoadhesin while internalisation and decapsidation of the virus require the trimer formed by viral GP2, GP3 and GP4 to interact with the host CD163.

**Fig. 1:**
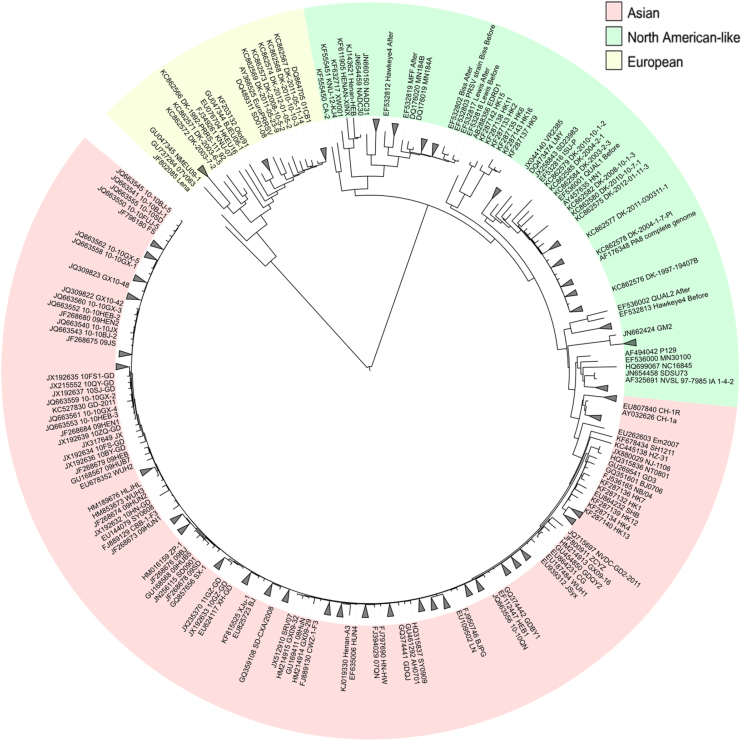
Phylogenetic analysis of whole genome PRRSV strains. Whole genome sequences from 336 PRRSV isolates currently available in the Genbanks were aligned with the program MUSCLE. The unrooted maximum likelihood phylogenetic tree with 500 bootstrap replications was constructed using MEGA5 and the tree drawn with iTOL. Accession number of each sequence precedes the isolate's name. The two main clades separate the virus into the European genotype 1 and North-American-like/Asian genotype 2.

PRRSV is a rapidly evolving pathogen (Murtaugh et al., 2010) with two predominant genotypes, the European Type 1 (Prototype strain Lelystad) and the North American-like Type 2 (Prototype strain VR-2332), which share approximately 60% nucleotide identity (Allende et al., 1999; Nelsen et al., 1999). However, current understanding from the findings of recent whole genome sequencing reveals the existence of considerably higher genetic variability making up a far more complex viral phylogenomics (Brar et al., 2014; Kvisgaard et al., 2013c; Zhou et al., 2014) (Fig. 1). Not only is genetic divergence evident between the two genotypes, individual PRRSV isolates and subtypes within each of the genotypes may vary up to approximately 20% at the nucleotide level (Han et al., 2006). For instance, variants of PRRSV Type 1 (subtypes 2, 3, and 4) identified in Eastern Europe (Stadejek et al., 2006, 2002) seem to be more virulent than the prototypical LV strain of PRRSV Type 1 subtype 1 in the pig model due probably to enhanced host cytokine activities (Morgan et al., 2013; Weesendorp et al., 2013). Moreover, other PRRSV Type 1 isolates with broader tropism capable of faster replication in the nasal mucosa have also emerged (Frydas et al., 2013). Similar to PRRSV Type 1, ongoing evolution within the PRRSV Type 2 genotype has been rather striking as well. In 1987, atypical PRRSV Type 2 strains were first identified in North America (Collins et al., 1992; Keffaber, 1989) but variant isolates/subtypes of these highly pathogenic PRRSV Type 2 strains have since spread to Asia (Li et al., 2007; Tian et al., 2007). Furthermore, microevolution within a coexisting quasispecies population has been found to further increase sequence heterogeneity in PRRSV (Goldberg et al., 2003). The dynamics of such mixed viral population is of increasing clinical importance with studies showing them affecting both virulence and pathogenesis (Domingo et al., 2012; Lauring and Andino, 2010).

An important manifestation of the PRRSV genomic complexity, especially within pig populations harbouring multiple circulating genotypes, is the resulting antigenic diversity (Liao et al., 2013). Therefore, control of PRRSV through the use of vaccines that offer cross protection among genetically variable genotypes remains challenging (Kimman et al., 2009). The phylogenomic insights revealed through the use of next generation sequencing (NGS) technologies may thus offer a better understanding of the relationship between the evolutionary dynamics and pathogenesis of PRRSV which in turn may aid in the design of more effective strategies to control the virus.

**Fig. 2:**
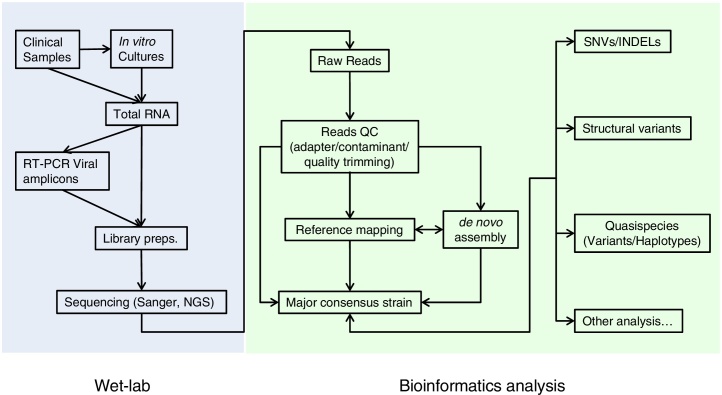
Proposed workflow for NGS analysis of PRRSV. The workflow includes a wet laboratory section where samples are prepared and sequenced; and a bioinformatics analysis section where raw sequencing data are pre-processed before undergoing various downstream analyses.

Here, we provide an introductory overview of the application of NGS to study the phylogenomics of PRRSV and we also suggest a general workflow (Fig. 2) to accomplish this. Although this workflow mainly focuses upon the use of the Illumina sequencing platforms, it should also be applicable to other NGS platforms with appropriate modifications.

## 2 Sequencing technologies

Conventional Sanger sequencing (Sanger et al., 1977) has over the last four decades revolutionised the entire field of biological research. The completion of the first human genome is one among the many important milestones achieved using this technology. The dideoxy or chain-termination process when coupled with automated fluorescent-labelling and capillary electrophoresis allows up to approximately 900 bp of nucleotides to be sequenced with high confidence. Although 96 samples can be sequenced in parallel, automated capillary sequencing remains relatively labour intensive, costly and time consuming; especially with regards to large-scale genome-wide studies.

With the introduction of NGS technologies about ten years ago, a new era of cost-effective and truly high-throughput genomic sciences was ushered in. The various NGS technologies that have been developed or are under development have been reviewed in detail elsewhere (Mardis, 2013; Metzker, 2010). Despite the differences in their underlying chemistries, sequencing protocols and throughputs, all current NGS workflows typically involve nucleic acid library preparation, DNA capturing follows by clonal amplification of individual molecules within the library and finally parallelised sequencing of the amplified library to yield, in the case of ultra-deep NGS, up to billions of sequencing reads. Depending on the platforms used, the 36 to ∼700 bp read lengths are shorter than those obtained with the conventional Sanger sequencing method. However, third generation sequencing technologies, exemplified by Pacific Biosciences and, potentially, Oxford Nanopore, have opened the door to read lengths of >10 kbp which go well beyond that obtained from Sanger sequencing (Morey et al., 2013).

Table 1 highlights and compares various features of Sanger sequencing against five of the most widely used high-throughput sequencing platforms that are currently available. In term of error rate, read length, yield, running cost and the intended applications, each of these NGS technologies has its own merits and weaknesses (Glenn, 2011; Quail et al., 2012; Ross et al., 2013). Nevertheless, all the leading NGS platforms are capable of running whole genome or transcriptome sequencing to a high degree of accuracy. On the other hand, for *de novo* assembly of either genomes/metagenomes or transcriptomes/metatranscriptomes, the longer read lengths from platforms such as 454, Ion Torrent, Illumina's Miseq and PacBio enable better resolution of low complexity regions and repeats. However, the numbers of reads produced by 454, Ion Torrent and PacBio may be insufficient for low cost full coverage and assembly of larger genomes. Where higher coverage is essential for cases such as detection of rare variants and haplotype reconstruction of viral quasispecies, the ultra-high throughput capacities of Illumina stands up among the other platforms. It is perhaps worth pointing out that for validation of variants detected using the NGS technologies, Sanger sequencing remains the preferred method since it is still regarded as the gold standard for its very low error rate (Ewing et al., 1998).

## 3 Application of NGS in PRRSV research

**Fig. 3:**
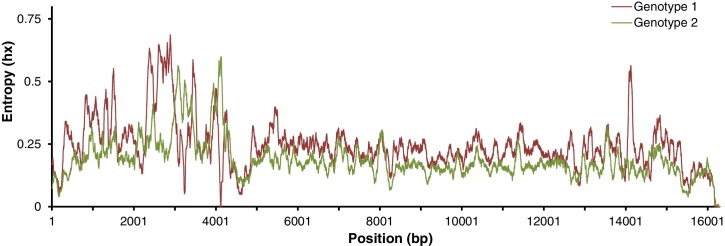
Sequence Entropy of whole genome PRRSV strains. Multiple whole genome sequence alignment of PRRSV genotype 1 and 2 was generated with MUSCLE and their respective entropy calculated with the molecular evolutionary program, Hyphy. The plots were constructed with 100 nt sliding window. In addition to common reported regions of variability, strains from PRRSV genotype 1 also exhibit higher degree of heterogeneity at the 3′ end of the genome.

Although PRRSV can be detected using antibody-based immunological methods, molecular methodologies such as PCR and sequencing remain the tools of choice for accurate identification of the virus at the genotypic or subgenotypic level. However, most previous studies on the genetic diversity of PPRSV have been restricted to the Sanger sequencing of the ORF5 and ORF7 which harbour the main immunogenic epitopes (Díaz et al., 2009; Martin-Valls et al., 2014; Oleksiewicz et al., 2002; Stadejek et al., 2013) and key diagnostic size polymorphisms resulted from insertion/deletion or recombination events (Stadejek et al., 2013). Although more than 12,000 sequences of the two ORFs have been deposited in the GenBank database to date, the drawback of such studies becomes immediately apparent when comparison of more than 300 whole genomes of PRRSV shows that there are clearly other variable regions in addition to just these two (Fig. 3). Indeed, variable regions unique to each of the two genotypes can also be identified. Therefore, phylogenomics studies based solely on partial PRRSV genomes may be misleading and the ability to sequence the whole PRRSV genomes at great depth makes NGS a powerful tool of choice. Indeed, in addition to genotyping, both conventional Sanger sequencing and NGS have been applied extensively to study various aspects of PRRSV biology. They include single gene/ORF discovery, whole genome transcriptomes, identification of subgenomic and heteroclite RNAs (Yuan et al., 2004), characterisation of viral diversity in terms of single nucleotide variants and recombination events, detection of viral quasispecies that evolve in response to immune pressure, and following of persistent infection (Barzon et al., 2013; Beerenwinkel and Zagordi, 2011; Brar et al., 2014; Capobianchi et al., 2013; Chang et al., 2002; McElroy et al., 2014; Radford et al., 2012).

The hallmark brute-force sequencing capacity of NGS when used on a small genome, such as that of PRRSV, can easily result in the genome being sequenced hundreds to tens of thousands times over. This so-called ultra-deep sequencing offers a powerful tool to detect rare occurring variants. In comparison to some of the previous quasispecies studies involving laborious RT-PCR-cloning-sequencing works focusing on only a particular subset of PRRSV's structural and non-structural genes (Goldberg et al., 2003; Schommer and Kleiboeker, 2006), the advantage of studying the whole genome using ultra-deep NGS is obvious. The additional heterogeneity brought about by the viral quasispecies can indeed provide valuable insights into the microevolutionary events within a mixed PRRSV population. For instance, these events allow the selection pressure to be followed during a vaccination trial.

Similar to all other modern genomic studies, application of NGS to PRRSV involves a workflow that combines both ‘wet’ (laboratory) and ‘dry’ (bioinformatics) activities (Fig. 2). Briefly, RNAs isolated from either tissue or culture-enriched materials, are reverse transcribed before being sequenced for further downstream bioinformatics analysis.

### 3.1 Sample preparation and sequencing

Sample assessment, enrichment of nucleic acids, and construction of sequencing library form the three integral preparatory steps prior to running any NGS application. While these procedures have been comprehensively reviewed (Head et al., 2014), the sample preparation required for the application of NGS to PRRSV depends on whether or not there is prior knowledge of the viral sequence. Table 2 describes the advantages and limits between the two approaches in preparing PRRSV samples for the generation of Illumina sequencing libraries.

Using known genomic sequences of PRRSV Type 1 and Type 2 available in public databases, Guo et al. (2011), Kvisgaard et al. (2013b) and Brar et al. (2014) have independently described a fast and robust method to generate by PCR sets of large overlapping amplicons of the whole PRRSV genome using degenerate primers targeting the two main PRRSV genotypes. Three different NGS platforms, namely Illumina, 454 and Ion Torrent, were used in these studies. The PCR-based enrichment of starting RNA materials enabled successful sequencing of the virus in a wide range of infected clinical tissue materials, including serum, lung and lymph node, even when the viral load was limited (Kvisgaard et al., 2013a). On the other hand, this approach usually requires time-consuming optimisation of the PCR procedures in order to amplify the longer amplicons. In addition, the highly heterogeneous PRRSV clearly poses a challenging obstacle for locating known conserved regions for which PCR primers can be designed. And the prerequisite of prior knowledge of conserved regions also rules out the ability of this methodology to pick up novel strains with mutated primer binding sites. Furthermore, complete coverage of the full genome with no ambiguity may not always be achievable. Of the three platforms tested, 454 with its longer read length allowed a near complete PRRSV genome to be obtained while Illumina gave a much higher read depth. The poorer performance of Ion Torrent was probably the result of its higher error rates in sequencing. It is perhaps worth pointing out that gap closure or extending into novel regions with methods such as nested-PCR, cloning and 5′-RACE may not be trivial. While PCR- and prior knowledge-based techniques allow for rapid diagnosis and genotyping of some PRRSV strains/isolates, heterogeneity of the virus may be seriously under-represented when variabilities and gaps outwith the known conserved regions and potential quasispecies within the same population are not included.

More recently, by bypassing the initial *in vitro* RT-PCR step and taking the advantage offered by the 225 bp longer read length from Illumina's Miseq machine, a complete PRRSV genome was successfully constructed without any prior knowledge of the viral sequence (Lu et al., 2014). This approach clearly offers the potential advantage of obtaining complete novel PRRSV sequences. However, it is important to note that a higher load of viral RNA was needed before the desired sequencing depth could be achieved. In addition, the “contaminating” host transcriptome, which was in fact the major species of nucleic acids in the sample, sequenced concurrently has to be removed. There is, of course, value in this “contaminating” host transcriptome sequences. When analysed, it provides an insight into potential host responses towards the PRRSV infection.

### 3.2 Bioinformatics analysis

#### 3.2.1 Pre-processing

Although not an essential step in the NGS analysis of eukaryotic or bacterial genomes, quality check or pre-processing of raw reads used in the mapping of fast evolving viral genomes is highly recommended. In spite of the much improved accuracy and precision of various NGS platforms, errors in raw data are not uncommon. They include remnant and/or read-through adaptors on both the 5′ and 3′ ends of sequencing reads; low quality (<*Q*_phred_ 20) and ambiguous bases; chimeric reads; platform-specific artefacts; long homopolymer stretches; and contaminating host nucleic acids (Meacham et al., 2011; Nakamura et al., 2011; Shao et al., 2013; Wang et al., 2012a). Additionally, the infidelity of polymerases, though at a very low rate, used during the amplification of the sample library is potentially another source of error. Ignoring or failing to sufficiently clean up these erroneous data may seriously compromise subsequent downstream biological inferences. Suboptimal mapping of these error containing reads against the reference genome may well lead to false identification of single nucleotide variants (SNVs) and/or indels and skew the estimation of the population complexity. Furthermore, any errors in the raw data can also unnecessarily complicate the *de novo* assembly process resulting in additional erroneous contigs being assembled. Not only is this going to increase the computational demands but more importantly, the resulting scaffolds may be too fragmented to allow the full construction of the novel genome. The multiplicity effects of any false positive bases or substitutions can indeed have a very profound impact on the accuracy of phylogenomic typing of small viral genomes. Not only can new strains be wrongly identified, the complexity of the quasispecies population may also be wrongly inflated.

Although different algorithms haven been applied to develop various pre-processing tools with diverse features, most of them deal specifically with issues related to the Illumina NGS reads; owing simply to the current dominance of the Illumina platform in the field (Hadfield and Loman, 2014). Many of these tools have been compared and reviewed elsewhere (Del Fabbro et al., 2013; Trivedi et al., 2014; Yang et al., 2013). They include fastx toolkits (Hannon, 2009), fastqc (Andrews, 2010), Cutadapt (Martin, 2011), Sickle (Joshi and Fass, 2011) and Scythe (Buffalo, 2011). While the types of the dataset, requirements of downstream analysis and various parameter-dependent trade-offs ultimately decide which programme is to be used, the quality-control/pre-processing procedure usually starts with initial quality plots of the raw data, followed by different trimming/filtering steps and finally the removal of contaminants. In the case with PRRSV, the most likely “contaminant” is from either the host's (*i.e.* pig) DNA or RNA which can subsequently be removed with mapping programs such as BWA (Li and Durbin, 2010), Bowtie (Langmead and Salzberg, 2012) or BMTagger (Rotmistrovsky and Agarwala, 2011).

#### 3.2.2 Mapping *vs de novo* assembly

To date, bioinformatics analyses of most of the recent PRRSV whole-genome sequencing using NGS technologies are limited to mapping the NGS reads to a one of the two prototypic reference PRRSV genomes. Among the many mapping tools developed, BWA and Bowtie are perhaps two of the most mature and widely used ones. The latter is known to run at a faster speed for shorter reads but BWA has a slight advantage in term of mapping accuracy (Li, 2013). While mapping is a fast and efficient way to identify new variants in closely related genomes, the default mapping stringency of these tools may prevent a significant number of real but less similar reads from being successfully mapped to the reference genome. The end result of such a mapping exercise is usually an incomplete consensus genome with variants of lower confidence. A *de novo* assembly should then be carried out to either close the gaps or identify potential novel recombination and mutation events.

*De novo* assembly is essentially the piecing together of a jigsaw puzzle involving millions or even billions of short NGS reads. Over the last few years, many draft genomes have been constructed to varying degrees of completeness using as many protocols and tools (Bradnam et al., 2013; Earl et al., 2011; Howison et al., 2013). Depending on the lengths of the NGS reads, these assembly tools are based broadly on either the long read overlapping or short read k-mer graph construction algorithm. It is important to note that regardless of the algorithms used, cleaned raw data of the highest quality is of absolute essential. Very often a hybrid approach using long and short NGS reads is necessary when highly divergence genomes are analysed (Bashir et al., 2012; Wang et al., 2012b). The quality of genomes assembled using the hybrid approach can then be checked by mapping the short reads back to the assembly.

#### 3.2.3 Downstream analysis

Consensus sequence of the mapped genome are routinely constructed by replacing the reference bases with SNVs/Indels identified using tools such as Samtools (Li et al., 2009) and GATK (McKenna et al., 2010). This sequence can then be compared against that derived from the *de novo* assembly to confirm the quality and accuracy of the novel genome derived from both mapping and assembly.

This final consensus sequence representative of the major strain in the viral population can then be used as a reference genome and fed into the mapping workflow using the same cleaned dataset. A more relaxed variant calling regime in Samtools/GATK or other low-frequency variant detecting tools, such as SNVer (Wei et al., 2011) and LoFreq (Wilm et al., 2012), can next be applied to search for the presence of variants arising from inter- or intra-population quasispecies at frequencies as low as 1% (Lu et al., 2014). At this low frequency, the ultra-deep sequence coverage can provide at least a few reads to confidently support the detected variants; lending more weight to the existence of the quasispecies. However, this approach provides only a combined spectrum of all variants in the total quasipecies population. Separation of co-occurring variants or local haplotype clustering and full construction of global haplotypes are necessary to reveal not only the true diversity of the virus but more importantly to allow a better phenotypic interpretation of the viral evolution in response to stresses such as vaccine or antiviral drug treatment. Although a few algorithms have been proposed, the disentanglement of the huge number of short NGS reads to reconstruct the true viral haplotypes remains an imperfect task; especially when analysing haplotypes present at low-frequency (Schirmer et al., 2014). Nevertheless, high quality longer reads from platform such as 454 and PacBio do in general provide better resolution. Indeed, complete genomes of a number of microorganisms with varying repeat contents have recently been assembled to their whole entirety using reads generated by the PacBio platform (Chin et al., 2013).

In addition to the detection of new variants and construction of novel genomes, read depth or coverage across the analysed genome can reveal other transcriptional features of PRRSV which may be difficult to uncover with conventional molecular biology methods. The higher than average coverage at the 5′ leader and 3′ UTR is indicative of active transcription of the genomic and subgenomic RNAs. The changes of coverage for each of the subgenomic RNAs at different time points can also help to further the understanding of the roles each of the transcribed genes play during the infection cycle.

The information gained from bioinformatics analysis of PRRSV's NGS studies is therefore multi-dimensional and indeed unprecedentedly insightful.

## 4 Future challenges and conclusion

Over the past years, NGS technologies have brought about a dramatic change in the genomic landscape of biology. Such technologies have become an essential tool in the field of viral infectious diseases and it is likely that PRRSV research and diagnosis will benefit greatly from the advancement of NGS in the years to come. For example, such technology could help shed light on the genetic evolution of PRRSV during immune selection (Evans et al., 2014). However, further developments are required for the field to gain even more from this NGS revolution. One of such development would be the ability to acquire, with minimal or no viral enrichment and without prior knowledge of any conserved regions, the full genomic sequence of PRRSV directly from clinical samples such as blood, serum, nasopharyngeal swabs or tissue biopsies. In fact, works are now underway in different laboratories to attempt to sequence full viral genomes directly from primary infected tissues (personal communication). It is anticipated that genomic data gained from such a direct tissue study can provide an unprecedented insight into PRRSV variants distribution within host-tissues and potentially unravelling novel PRRSV's subgenomic, heteroclite and other probable RNA species. However, past studies have also highlighted the need to develop PCR- and culture-free methods to enrich the viral RNA in order to increase the sequencing depth to a level where rare variants from quasispecies can be detected confidently. Perhaps, methods involving sequence-independent amplification such as capture-by-hybridisation (Bent et al., 2013; Kwok et al., 2014) could take full advantage of the *ca* ∼370 currently available full genomes of PRRSV. In addition, although ultra-deep NGS has enabled the calling of low frequency variants using sophisticated bioinformatics tools, challenges remain to be overcome for accurate haplotype reconstruction of viral variants present in either very low number or with structural variations. Promisingly, however, it is likely that the advent of third generation sequencing technologies capable of long-read single-molecule sequencing without prior amplification, such as the those offered by PacBio and Oxford Nanapore, may enable not only assembly of genomes with high accuracy but also the construction of whole genome quasispecies (Giallonardo et al., 2014).

Genomics data obtained using NGS can provide valuable insights into features of viral populations, such as viral attenuation, response to host immune pressure, disease severity and transmission, which are currently masked by other measures. Taken together it is likely that the use of NGS on routine basis will allow the development of rapid and tailored responses to combat the elusive PRRS disease.

## Figures and Tables

**Table 1:** Comparison of conventional Sanger and NGS technologies.

Platform (vendor)	Technology	Run time	Read length per run (bp)	Max. yield	Final per base error rate (%)
Sanger (Applied Biosystems)	Chain termination	0.5–3 h	∼700–900	∼86 kbp	0.001–1.0
HiSeq/Miseq (Illumina)	Solid-phase PCR/reversible chain termination	4 h–11 days	36–300	15–1800 Gbp	∼0.1
454 (Roche)	emPCR^a^/pyro sequencing	10–20 h	∼400–700	700 Mbp	∼1
SOLID (Life Technologies)	emPCR/sequencing by ligation & 2-base coding	8 days	85–110	155 Gbp	≤0.1
Ion Torrent (Life Technologies)	emPCR/semiconductor sequencing	2.5–7.5 h	175–400	12 Gbp	∼2
PacBio (Pacific Biosciences)	SMRT^b^ sequencing	∼0.5–3 h	∼8500	∼375 Mbp/cell	≤1

^a^ emPCR: emulsion PCR. ^b^ SMRT: single molecule, real-time.

**Table 2:** Sample preparations and aims.

	With prior knowledge of PRRSV sequence	Without prior knowledge of PRRSV sequence
Viral titre	Not necessarily high	High
PCR amplification and optimisation	Yes	No
Virus/RNA enrichment	No	Often required
Host sequence contamination	No	Often very high
Multiplexing of strains	Yes	Yes
Full genome	Not always	Yes
Read depth	High	Variable
Viral transcriptome	No	Yes
References	Brar et al. (2014), Kvisgaard et al. (2013b)	Lu et al. (2014)

## References

[bib0005] Allende R., Lewis T.L., Lu Z., Rock D.L., Kutish G.F., Ali A., Doster A.R., Osorio F.A. (1999). North American and European porcine reproductive and respiratory syndrome viruses differ in non-structural protein coding regions. J. Gen. Virol..

[bib0010] Andrews S. (2010). FastQC: A Quality Control Tool for High Throughput Sequence Data. http://www.bioinformatics.babraham.ac.uk/projects/fastqc/.

[bib0015] Barzon L., Lavezzo E., Costanzi G., Franchin E., Toppo S., Palu G. (2013). Next-generation sequencing technologies in diagnostic virology. J. Clin. Virol..

[bib0020] Bashir A., Klammer A.A., Robins W.P., Chin C.S., Webster D., Paxinos E., Hsu D., Ashby M., Wang S., Peluso P., Sebra R., Sorenson J., Bullard J., Yen J., Valdovino M., Mollova E., Luong K., Lin S., LaMay B., Joshi A., Rowe L., Frace M., Tarr C.L., Turnsek M., Davis B.M., Kasarskis A., Mekalanos J.J., Waldor M.K., Schadt E.E. (2012). A hybrid approach for the automated finishing of bacterial genomes. Nat. Biotechnol..

[bib0025] Beerenwinkel N., Zagordi O. (2011). Ultra-deep sequencing for the analysis of viral populations. Curr. Opin. Virol..

[bib0030] Bent Z.W., Tran-Gyamfi M.B., Langevin S.A., Brazel D.M., Hamblin R.Y., Branda S.S., Patel K.D., Lane T.W., VanderNoot V.A. (2013). Enriching pathogen transcripts from infected samples: a capture-based approach to enhanced host–pathogen RNA sequencing. Anal. Biochem..

[bib0035] Bradnam K.R., Fass J.N., Alexandrov A., Baranay P., Bechner M., Birol I., Boisvert S., Chapman J.A., Chapuis G., Chikhi R., Chitsaz H., Chou W.C., Corbeil J., Del Fabbro C., Docking T.R., Durbin R., Earl D., Emrich S., Fedotov P., Fonseca N.A., Ganapathy G., Gibbs R.A., Gnerre S., Godzaridis E., Goldstein S., Haimel M., Hall G., Haussler D., Hiatt J.B., Ho I.Y., Howard J., Hunt M., Jackman S.D., Jaffe D.B., Jarvis E.D., Jiang H., Kazakov S., Kersey P.J., Kitzman J.O., Knight J.R., Koren S., Lam T.W., Lavenier D., Laviolette F., Li Y., Li Z., Liu B., Liu Y., Luo R., Maccallum I., Macmanes M.D., Maillet N., Melnikov S., Naquin D., Ning Z., Otto T.D., Paten B., Paulo O.S., Phillippy A.M., Pina-Martins F., Place M., Przybylski D., Qin X., Qu C., Ribeiro F.J., Richards S., Rokhsar D.S., Ruby J.G., Scalabrin S., Schatz M.C., Schwartz D.C., Sergushichev A., Sharpe T., Shaw T.I., Shendure J., Shi Y., Simpson J.T., Song H., Tsarev F., Vezzi F., Vicedomini R., Vieira B.M., Wang J., Worley K.C., Yin S., Yiu S.M., Yuan J., Zhang G., Zhang H., Zhou S., Korf I.F. (2013). Assemblathon 2- evaluating de novo methods of genome assembly in three vertebrate species. GigaScience.

[bib0040] Brar M.S., Shi M., Hui R.K., Leung F.C. (2014). Genomic evolution of porcine reproductive and respiratory syndrome virus (PRRSV) isolates revealed by deep sequencing. PLOS ONE.

[bib0045] Brockmeier S.L., Loving C.L., Vorwald A.C., Kehrli M.E., Baker R.B., Nicholson T.L., Lager K.M., Miller L.C., Faaberg K.S. (2012). Genomic sequence and virulence comparison of four type 2 porcine reproductive and respiratory syndrome virus strains. Virus Res..

[bib0050] Buffalo V. (2011). Scythe – A Bayesian Adapter Trimmer. http://github.com/vsbuffalo/scythe.

[bib0055] Capobianchi M.R., Giombini E., Rozera G. (2013). Next-generation sequencing technology in clinical virology. Clin. Microbiol. Infect..

[bib0060] Chang C.C., Yoon K.J., Zimmerman J.J., Harmon K.M., Dixon P.M., Dvorak C.M., Murtaugh M.P. (2002). Evolution of porcine reproductive and respiratory syndrome virus during sequential passages in pigs. J. Virol..

[bib0065] Chin C.S., Alexander D.H., Marks P., Klammer A.A., Drake J., Heiner C., Clum A., Copeland A., Huddleston J., Eichler E.E., Turner S.W., Korlach J. (2013). Nonhybrid, finished microbial genome assemblies from long-read SMRT sequencing data. Nat. Methods.

[bib0070] Collins J.E., Benfield D.A., Christianson W.T., Harris L., Hennings J.C., Shaw D.P., Goyal S.M., McCullough S., Morrison R.B., Joo H.S. (1992). Isolation of swine infertility and respiratory syndrome virus (isolate ATCC VR-2332) in North America and experimental reproduction of the disease in gnotobiotic pigs. J. Vet. Diagn. Invest.: Off. Publ. Am. Assoc. Vet. Lab. Diagn. Inc..

[bib0075] Del Fabbro C., Scalabrin S., Morgante M., Giorgi F.M. (2013). An extensive evaluation of read trimming effects on Illumina NGS data analysis. PLOS ONE.

[bib0080] Díaz I., Pujols J., Ganges L., Gimeno M., Darwich L., Domingo M., Mateu E. (2009). In silico prediction and ex vivo evaluation of potential T-cell epitopes in glycoproteins 4 and 5 and nucleocapsid protein of genotype-I (European) of porcine reproductive and respiratory syndrome virus. Vaccine.

[bib0085] Domingo E., Sheldon J., Perales C. (2012). Viral quasispecies evolution. Microbiol. Mol. Biol. Rev..

[bib0090] Earl D., Bradnam K., St John J., Darling A., Lin D., Fass J., Yu H.O., Buffalo V., Zerbino D.R., Diekhans M., Nguyen N., Ariyaratne P.N., Sung W.K., Ning Z., Haimel M., Simpson J.T., Fonseca N.A., Birol I., Docking T.R., Ho I.Y., Rokhsar D.S., Chikhi R., Lavenier D., Chapuis G., Naquin D., Maillet N., Schatz M.C., Kelley D.R., Phillippy A.M., Koren S., Yang S.P., Wu W., Chou W.C., Srivastava A., Shaw T.I., Ruby J.G., Skewes-Cox P., Betegon M., Dimon M.T., Solovyev V., Seledtsov I., Kosarev P., Vorobyev D., Ramirez-Gonzalez R., Leggett R., MacLean D., Xia F., Luo R., Li Z., Xie Y., Liu B., Gnerre S., MacCallum I., Przybylski D., Ribeiro F.J., Yin S., Sharpe T., Hall G., Kersey P.J., Durbin R., Jackman S.D., Chapman J.A., Huang X., DeRisi J.L., Caccamo M., Li Y., Jaffe D.B., Green R.E., Haussler D., Korf I., Paten B. (2011). Assemblathon 1: a competitive assessment of de novo short read assembly methods. Genome Res..

[bib0095] Evans A.B., Dorman K.S., King E.A., Lunney J.K., Rowland R.R.R., Carpenter S. (2014). Longitudinal analyses of viral genotypes during experimental PRRSV infection suggest rebound, in viremia is due to antigenic variation and immune escape. Nidovirus.

[bib0100] Ewing B., Hillier L., Wendl M.C., Green P. (1998). Base-calling of automated sequencer traces using phred I. Accuracy assessment. Genome Res..

[bib0105] Frydas I.S., Verbeeck M., Cao J., Nauwynck H.J. (2013). Replication characteristics of porcine reproductive and respiratory syndrome virus (PRRSV) European subtype 1 (Lelystad) and subtype 3 (Lena) strains in nasal mucosa and cells of the monocytic lineage: indications for the use of new receptors of PRRSV (Lena). Vet. Res..

[bib0110] Giallonardo F.D., Topfer A., Rey M., Prabhakaran S., Duport Y., Leemann C., Schmutz S., Campbell N.K., Joos B., Lecca M.R., Patrignani A., Daumer M., Beisel C., Rusert P., Trkola A., Gunthard H.F., Roth V., Beerenwinkel N., Metzner K.J. (2014). Full-length haplotype reconstruction to infer the structure of heterogeneous virus populations. Nucleic Acids Res..

[bib0115] Glenn T. (2011). Field guide to next-generation DNA sequencers. Mol. Ecol. Resour..

[bib0120] Goldberg T.L., Lowe J.F., Milburn S.M., Firkins L.D. (2003). Quasispecies variation of porcine reproductive and respiratory syndrome virus during natural infection. Virology.

[bib0125] Guo B., Vorwald A.C., Alt D.P., Lager K.M., Bayles D.O., Faaberg K.S. (2011). Large scale parallel pyrosequencing technology: PRRSV strain VR-2332 nsp2 deletion mutant stability in swine. Virus Res..

[bib0130] Hadfield J., Loman N. (2014). Next Generation Genomics: World Map of High-Throughput Sequencers. http://omicsmaps.com/.

[bib0135] Han J., Wang Y., Faaberg K.S. (2006). Complete genome analysis of RFLP 184 isolates of porcine reproductive and respiratory syndrome virus. Virus Res..

[bib0140] Hannon G. (2009). FASTX-Toolkit. http://hannonlab.cshl.edu/fastx_toolkit/index.html.

[bib0145] Head S.R., Komori H.K., LaMere S.A., Whisenant T., Van Nieuwerburgh F., Salomon D.R., Ordoukhanian P. (2014). Library construction for next-generation sequencing: overviews and challenges. BioTechniques.

[bib0150] Howison M., Zapata F., Dunn C.W. (2013). Toward a statistically explicit understanding of de novo sequence assembly. Bioinformatics.

[bib0155] Joshi N., Fass J. (2011). Sickle – A Windowed Adaptive Trimming Tool for FASTQ Files Using Quality. http://github.com/najoshi/sickle.

[bib0160] Keffaber K.K. (1989). Reproductive failure of unknown etiology. Am. Assoc. Swine Pract. Newsl..

[bib0165] Kimman T.G., Cornelissen L.A., Moormann R.J., Rebel J.M., Stockhofe-Zurwieden N. (2009). Challenges for porcine reproductive and respiratory syndrome virus (PRRSV) vaccinology. Vaccine.

[bib0170] Kvisgaard L.K., Hjulsager C.K., Brar M.S., Leung F.C., Larsen L.E. (2013). Genetic dissection of complete genomes of Type 2 PRRS viruses isolated in Denmark over a period of 15 years. Vet. Microbiol..

[bib0175] Kvisgaard L.K., Hjulsager C.K., Fahnøe U., Breum S.Ø., Ait-Ali T., Larsen L.E. (2013). A fast and robust method for full genome sequencing of Porcine Reproductive and Respiratory Syndrome Virus (PRRSV) Type 1 and Type 2. J. Virol. Methods.

[bib0180] Kvisgaard L.K., Hjulsager C.K., Kristensen C.S., Lauritsen K.T., Larsen L.E. (2013). Genetic and antigenic characterization of complete genomes of Type 1 Porcine Reproductive and Respiratory Syndrome viruses (PRRSV) isolated in Denmark over a period of 10 years. Virus Res..

[bib0185] Kwok H., Wu C.W., Palser A.L., Kellam P., Sham P.C., Kwong D.L., Chiang A.K. (2014). Genomic diversity of Epstein–Barr virus genomes isolated from primary nasopharyngeal carcinoma biopsy samples. J. Virol..

[bib0190] Langmead B., Salzberg S.L. (2012). Fast gapped-read alignment with Bowtie 2. Nat. Methods.

[bib0195] Lauring A.S., Andino R. (2010). Quasispecies theory and the behavior of RNA viruses. PLoS Path..

[bib0200] Li H. (2013). Aligning sequence reads, clone sequences and assembly contigs with BWA-MEM. ArXiv e-prints.

[bib0205] Li H., Durbin R. (2010). Fast and accurate long read alignment with Burrows–Wheeler transform. Bioinformatics.

[bib0210] Li H., Handsaker B., Wysoker A., Fennell T., Ruan J., Homer N., Marth G., Abecasis G., Durbin R., Genome Project Data Processing S (2009). The Sequence Alignment/Map format and SAMtools. Bioinformatics.

[bib0215] Li Y., Wang X., Bo K., Wang X., Tang B., Yang B., Jiang W., Jiang P. (2007). Emergence of a highly pathogenic porcine reproductive and respiratory syndrome virus in the Mid-Eastern region of China. Vet. J..

[bib0220] Liao Y.C., Lin H.H., Lin C.H., Chung W.B. (2013). Identification of cytotoxic T lymphocyte epitopes on swine viruses: multi-epitope design for universal T cell vaccine. PLOS ONE.

[bib0225] Lu Z.H., Brown A., Wilson A.D., Calvert J.G., Balasch M., Fuentes-Utrilla P., Loecherbach J., Turner F., Talbot R., Archibald A.L., Ait-Ali T. (2014). Genomic variation in macrophage-cultured European porcine reproductive and respiratory syndrome virus Olot/91 revealed using ultra-deep next generation sequencing. Virol. J..

[bib0230] Mardis E.R. (2013). Next-generation sequencing platforms. Annu. Rev. Anal. Chem..

[bib0235] Martin-Valls G.E., Kvisgaard L.K., Tello M., Darwich L., Cortey M., Burgara-Estrella A.J., Hernandez J., Larsen L.E., Mateu E. (2014). Analysis of ORF5 and full-length genome sequences of porcine reproductive and respiratory syndrome virus isolates of genotypes 1 and 2 retrieved worldwide provides evidence that recombination is a common phenomenon and may produce mosaic isolates. J. Virol..

[bib0240] Martin M. (2011). Cutadapt removes adapter sequences from high-throughput sequencing reads. EMBnet.journal.

[bib0245] McElroy K., Thomas T., Luciani F. (2014). Deep sequencing of evolving pathogen populations: applications, errors, and bioinformatic solutions. Microb. Inform. Exp..

[bib0250] McKenna A., Hanna M., Banks E., Sivachenko A., Cibulskis K., Kernytsky A., Garimella K., Altshuler D., Gabriel S., Daly M., DePristo M.A. (2010). The genome analysis toolkit: a MapReduce framework for analyzing next-generation DNA sequencing data. Genome Res..

[bib0255] Meacham F., Boffelli D., Dhahbi J., Martin D., Singer M., Pachter L. (2011). Identification and correction of systematic error in high-throughput sequence data. BMC Bioinform..

[bib0260] Metzker M.L. (2010). Sequencing technologies – the next generation. Nat. Rev. Genet..

[bib0265] Meulenberg J.J.M., Hulst M.M., de Meijer E.J., Moonen P.L.J.M., den Besten A., de Kluyver E.P., Wensvoort G., Moormann R.J.M. (1993). Lelystad virus, the causative agent of Porcine Epidemic Abortion and Respiratory Syndrome (PEARS), is related to LDV and EAV. Virology.

[bib0270] Morey M., Fernandez-Marmiesse A., Castineiras D., Fraga J.M., Couce M.L., Cocho J.A. (2013). A glimpse into past, present, and future DNA sequencing. Mol. Genet. Metab..

[bib0275] Morgan S.B., Graham S.P., Salguero F.J., Sánchez Cordón P.J., Mokhtar H., Rebel J.M.J., Weesendorp E., Bodman-Smith K.B., Steinbach F., Frossard J.P. (2013). Increased pathogenicity of European porcine reproductive and respiratory syndrome virus is associated with enhanced adaptive responses and viral clearance. Vet. Microbiol..

[bib0280] Murtaugh M.P., Stadejek T., Abrahante J.E., Lam T.T., Leung F.C. (2010). The ever-expanding diversity of porcine reproductive and respiratory syndrome virus. Virus Res..

[bib0285] Nakamura K., Oshima T., Morimoto T., Ikeda S., Yoshikawa H., Shiwa Y., Ishikawa S., Linak M.C., Hirai A., Takahashi H., Altaf-Ul-Amin M., Ogasawara N., Kanaya S. (2011). Sequence-specific error profile of Illumina sequencers. Nucleic Acids Res..

[bib0290] Nelsen C.J., Murtaugh M.P., Faaberg K.S. (1999). Porcine Reproductive and Respiratory Syndrome virus comparison: divergent evolution on two continents. J. Virol..

[bib0295] Neumann E.J., Kliebenstein J.B., Johnson C.D., Mabry J.W., Bush E.J., Seitzinger A.H., Green A.L., Zimmerman J.J. (2005). Assessment of the economic impact of porcine reproductive and respiratory syndrome on swine production in the United States. J. Am. Vet. Med. Assoc..

[bib0300] Nieuwenhuis N., Duinhof T.F., van Nes A. (2012). Economic analysis of outbreaks of porcine reproductive and respiratory syndrome virus in nine sow herds. Vet. Rec..

[bib0305] Oleksiewicz M.B., Bøtner A., Normann P. (2002). Porcine B-cells recognize epitopes that are conserved between the structural proteins of American- and European-type porcine reproductive and respiratory syndrome virus. J. Gen. Virol..

[bib0310] Quail M.A., Smith M., Coupland P., Otto T.D., Harris S.R., Connor T.R., Bertoni A., Swerdlow H.P., Gu Y. (2012). A tale of three next generation sequencing platforms: comparison of Ion Torrent, Pacific Biosciences and Illumina MiSeq sequencers. BMC Genomics.

[bib0315] Radford A.D., Chapman D., Dixon L., Chantrey J., Darby A.C., Hall N. (2012). Application of next-generation sequencing technologies in virology. J. Gen. Virol..

[bib0320] Ross M.G., Russ C., Costello M., Hollinger A., Lennon N.J., Hegarty R., Nusbaum C., Jaffe D.B. (2013). Characterizing and measuring bias in sequence data. Genome Biol..

[bib0325] Rotmistrovsky K., Agarwala R. (2011). BMTagger: Best Match Tagger for Removing Human Reads from Metagenomics Datasets. ftp://ftp.ncbi.nlm.nih.gov/pub/agarwala/bmtagger/.

[bib0330] Sanger F., Nicklen S., Coulson A.R. (1977). DNA sequencing with chain-terminating inhibitors. Proc. Natl. Acad. Sci. U. S. A.

[bib0335] Schirmer M., Sloan W.T., Quince C. (2014). Benchmarking of viral haplotype reconstruction programmes: an overview of the capacities and limitations of currently available programmes. Brief. Bioinform..

[bib0340] Schommer S.K., Kleiboeker S.B. (2006). Use of a PRRSV infectious clone to evaluate in vitro quasispecies evolution. Adv. Exp. Med. Biol..

[bib0345] Shao W., Boltz V.F., Spindler J.E., Kearney M.F., Maldarelli F., Mellors J.W., Stewart C., Volfovsky N., Levitsky A., Stephens R.M., Coffin J.M. (2013). Analysis of 454 sequencing error rate, error sources, and artifact recombination for detection of Low-frequency drug resistance mutations in HIV-1 DNA. Retrovirology.

[bib0350] Shi M., Lam T.T., Hon C.C., Hui R.K., Faaberg K.S., Wennblom T., Murtaugh M.P., Stadejek T., Leung F.C. (2010). Molecular epidemiology of PRRSV: a phylogenetic perspective. Virus Res..

[bib0355] Snijder E.J., Kikkert M., Fang Y. (2013). Arterivirus molecular biology and pathogenesis. J. Gen. Virol..

[bib0360] Snijder E.J., Meulenberg J.J. (1998). The molecular biology of arteriviruses. J. Gen. Virol..

[bib0365] Stadejek T., Oleksiewicz M.B., Potapchuk D., Podgorska K. (2006). Porcine reproductive and respiratory syndrome virus strains of exceptional diversity in eastern Europe support the definition of new genetic subtypes. J. Gen. Virol..

[bib0370] Stadejek T., Stankevicius A., Murtaugh M.P., Oleksiewicz M.B. (2013). Molecular evolution of PRRSV in Europe: current state of play. Vet. Microbiol..

[bib0375] Stadejek T., Stankevicius A., Storgaard T., Oleksiewicz M.B., Belak S., Drew T.W., Pejsak Z. (2002). Identification of radically different variants of porcine reproductive and respiratory syndrome virus in Eastern Europe: towards a common ancestor for European and American viruses. J. Gen. Virol..

[bib0380] Tian K., Yu X., Zhao T., Feng Y., Cao Z., Wang C., Hu Y., Chen X., Hu D., Tian X., Liu D., Zhang S., Deng X., Ding Y., Yang L., Zhang Y., Xiao H., Qiao M., Wang B., Hou L., Wang X., Yang X., Kang L., Sun M., Jin P., Wang S., Kitamura Y., Yan J., Gao G.F. (2007). Emergence of fatal PRRSV variants: unparalleled outbreaks of atypical PRRS in China and molecular dissection of the unique hallmark. PLoS ONE.

[bib0385] Trivedi U.H., Cezard T., Bridgett S., Montazam A., Nichols J., Blaxter M., Gharbi K. (2014). Quality control of next-generation sequencing data without a reference. Front. Genet..

[bib0390] Van Breedam W., Delputte P.L., Van Gorp H., Misinzo G., Vanderheijden N., Duan X., Nauwynck H.J. (2010). Porcine reproductive and respiratory syndrome virus entry into the porcine macrophage. J. Gen. Virol..

[bib0395] Wang X., Blades N., Ding J., Sultana R., Parmigiani G. (2012). Estimation of sequencing error rates in short reads. BMC Bioinform..

[bib0400] Wang Y., Yu Y., Pan B., Hao P., Li Y., Shao Z., Xu X., Li X. (2012). Optimizing hybrid assembly of next-generation sequence data from *Enterococcus faecium*: a microbe with highly divergent genome. BMC Syst. Biol..

[bib0405] Weesendorp E., Morgan S., Stockhofe-Zurwieden N., Popma-De Graaf D.J., Graham S.P., Rebel J.M. (2013). Comparative analysis of immune responses following experimental infection of pigs with European porcine reproductive and respiratory syndrome virus strains of differing virulence. Vet. Microbiol..

[bib0410] Wei Z., Wang W., Hu P., Lyon G.J., Hakonarson H. (2011). SNVer: a statistical tool for variant calling in analysis of pooled or individual next-generation sequencing data. Nucleic Acids Res..

[bib0415] Wensvoort G., Terpstra C., Pol J.M., ter Laak E.A., Bloemraad M., de Kluyver E.P., Kragten C., van Buiten L., den Besten A., Wagenaar F. (1991). Mystery swine disease in The Netherlands: the isolation of Lelystad virus. Vet. Q..

[bib0420] Wilm A., Aw P.P., Bertrand D., Yeo G.H., Ong S.H., Wong C.H., Khor C.C., Petric R., Hibberd M.L., Nagarajan N. (2012). LoFreq: a sequence-quality aware, ultra-sensitive variant caller for uncovering cell-population heterogeneity from high-throughput sequencing datasets. Nucleic Acids Res..

[bib0425] Yang X., Chockalingam S.P., Aluru S. (2013). A survey of error-correction methods for next-generation sequencing. Brief. Bioinform..

[bib0430] Yuan S., Murtaugh M.P., Schumann F.A., Mickelson D., Faaberg K.S. (2004). Characterization of heteroclite subgenomic RNAs associated with PRRSV infection. Virus Res..

[bib0435] Zhou L., Yang X., Tian Y., Yin S., Geng G., Ge X., Guo X., Yang H. (2014). Genetic diversity analysis of genotype 2 porcine reproductive and respiratory syndrome viruses emerging in recent years in China. Biomed. Res. Int..

[bib0440] Zimmerman J.J., Yoon K.J., Wills R.W., Swenson S.L. (1997). General overview of PRRSV: a perspective from the United States. Vet. Microbiol..

